# Synthesis, Topological, and Biological Studies of a Novel One‐Dimensional Hg(II) Coordination Polymer With Antibacterial and Anticancer Potentials

**DOI:** 10.1155/bca/2319593

**Published:** 2026-01-31

**Authors:** Shekufeh Alaei, Khosro Mohammadi, Payam Hayati, Somayyeh Gharibi, Arezoo Khoradmehr, Zeinab Asen, Jan Janczak, Eugeny V. Alexandrov

**Affiliations:** ^1^ Department of Chemistry, Faculty of Nano and Bio Science and Technology, Persian Gulf University, Bushehr, 75169, Iran, pgu.ac.ir; ^2^ Department of Biotechnology and Life Sciences, University of Insubria, Via Jean Henry Dunant 3, Varese, 21100, Italy, uninsubria.eu; ^3^ Persian Gulf Marine Biotechnology Research Sciences Center, The Persian Gulf Biomedical Research Sciences Institute, Bushehr University of Medical Sciences, Bushehr, 75147, Iran, bpums.ac.ir; ^4^ Shamim beh Azma Company, Bushehr, Iran; ^5^ Institute of Low Temperature and Structure Research, Polish Academy of Sciences, Okolna 2 Street, Wroclaw, 50-422, Poland, pan.pl; ^6^ Center NTI “Digital Materials Science: New Materials and Substances”, Bauman Moscow State Technical University, 2nd Baumanskaya Street 5/1, Moscow, 105005, Russia, bmstu.ru

**Keywords:** 1D framework, antibacterial activity, anticancer, coordination polymer, isonicotinic acid, mercury (II)

## Abstract

A novel one‐dimensional mercury coordination polymer (CP), identified as [(μ_2_‐Cl)(Ina)Hg(μ_3_‐Cl)Hg(μ_2_‐Cl)_2_(Ina)]_n_ (**1**) (where Ina represents isonicotinic acid or 4‐pyridinecarboxylic acid), was synthesized via the interaction of isonicotinic acid with mercury(II) salt. This synthesis was achieved through two distinct experimental approaches: layering methods for the formation of single crystals (**1**) and sonochemical irradiation for the production of nanostructures (**1**′). The structural characterization of (**1**) was performed using X‐ray diffraction and crystallography techniques. Further characterization involved a range of methods, including X‐ray powder diffraction (XRD), infrared (IR) spectroscopy, scanning electron microscopy (SEM), thermogravimetric analysis (TGA), and Hirshfeld surface analysis (HSA). The CP of (**1**) features two types of metal centers, exhibiting coordination numbers of 5 and 6. In this structure, each mercury atom is coordinated to chlorine, nitrogen, and oxygen atoms derived from the ligands. Additionally, antibacterial properties were tested on seven Gram‐positive bacteria and nine Gram‐negative bacteria. Anticancer properties were tested on both OCAR3 (cancer) and VERO (normal) cells; as a result, the antibacterial and anticancer activities of nanoparticle [(μ_2_‐Cl)(Ina)Hg(μ_3_‐Cl)Hg(μ_2_‐Cl)_2_(Ina)]_n_ (**1**′) were evaluated, revealing that the antibacterial efficacy of the nanoparticles was comparable to that of standard antibiotics. The anticancer properties were effective in destroying cancer cells while preserving the integrity of normal cells. Consequently, both antibacterial and anticancer properties demonstrated promising results.

## 1. Introduction

Metalorganic coordination polymers (MOCPs) have attracted significant interest in recent years across multiple scientific disciplines [1]. This interest is primarily due to a variety of structural forms as well as the promising applications they offer. The distinctive integration of metal ions and organic ligands enables researchers to engineer materials with tailored properties, rendering them useful for a range of applications, including energy storage and environmental remediation [2]. Coordination polymers (CPs) represent a compelling category of materials, consisting of metal centers interconnected by organic ligands. This is able to be visualized as a network where the metal centers serve as nodes, while the ligands function as the connecting “bridges” [3]. CPs are solid‐state materials that have a repeating structure of coordination units. CPs extend in one, two, and three dimensions [4]. The first preparation and application of coordination compounds date back to antiquity, the early 18th century, when German chemists accidentally discovered Prussian blue dye [5].

The structure of CPs can be varied nearly at every step. The structure is generally made up through a judicious choice of metal ions and organic linkers, and a nearly limitless number of possible architectures can be obtained [6]. The CPs have some functional properties. The properties include store gases [7], separate gas [8], liquid [9] mixtures, water purification [10] activities, and biomedical applications [11].

The initial development of CPs was achieved through the utilization of inorganic complexes. Notable early instances include Hofmann clathrates, Prussian blue, and various cyanide complexes. These materials served as crucial foundations in the evolution of coordination chemistry, enhancing our comprehension of molecular arrangements in complex and repetitive structures [12, 13]. The term self‐assembly describes the phenomenon in which the constituent elements of a structure engage with one another to form a CP, which subsequently expands into larger polymeric formations across 1, 2, or even 3 dimensions [14]. Within CPs, the constituent elements, known as structural units, are interconnected by coordination bonds alongside weaker interactions such as hydrogen bonds, π–π stacking, and van der Waals forces [15]. Over time, these interactions facilitate the polymer’s growth in both size and complexity.

Subsequent reviews have concentrated on specific application areas of 1D CPs, such as high‐energy materials: investigating the role of 1D CPs in energy storage and release mechanism [16], spin‐crossover and valence tautomerism: exploring the unique electronic properties of 1D CPs that enable transitions between different oxidation states [17, 18], white‐light emissive materials: developing 1D CPs for applications in lighting and display technologies [19], electro functional complexes: designing 1D CPs with specific electrochemical properties for using in sensors and actuators [20], magnetism: studying the magnetic behaviors of 1D CPs for potential applications in data storage and spintronics [21–23], luminescence: exploring the light‐emitting properties of 1D CPs for applications in optoelectronics and bioimaging [24], energy storage: investigating the use of 1D CPs in batteries and supercapacitors for efficient energy storage solution [25], and catalysis: utilizing 1D CPs as catalysts in various chemical reactions, including organic transformations and environmental remediation [26]. Despite these focused studies, there has been limited research comprehensively addressing the various ligand types used in 1D CPs. Understanding the role of different ligands is crucial, as they significantly influence the structural and functional properties of these materials. Further exploration in this area could lead to the development of more versatile and efficient 1D CPs for a wide range of applications.

Mercury has historically played a crucial role in various pharmaceutical formulations, such as laxatives, antiseptics, diuretics, and antibacterial compounds [27]. Nevertheless, it has become evident that prolonged mercury exposure can adversely affect human health, particularly the nervous system [28]. In recent years, mercury (II) ions (Hg^2+^) have attracted considerable attention within the field of supramolecular coordination chemistry, particularly over the last 20 years. This interest stems from their capacity to create CPs characterized by a wide array of flexible geometries. These geometries include trigonal, tetragonal, square pyramidal, and octahedral forms, which correspond to coordination numbers ranging from 2 to 6 [29]. The mercury complexes prove their efficiency in acting effectively either for treatment purposes or for diagnosis in biomedicine research. Our success in applying mercury complexes in medical practice necessitates advances in their stability properties and toxicities [30]. Mercury complexes are amongst the substances whose biological activities are taken into consideration [31]. Although the toxicity of mercury is harmful to some organs in the body, bioinorganic chemists have recently demonstrated positive biological properties in some mercury complexes. Mercury, owing to its toxicity as well as its unique position among other elements, can be proposed as an element of interest in the design of antimicrobial compounds [32]. El‐Megharbel and Refat demonstrated the antibacterial effectiveness of Hg(II) complexes of Fe(III), Ag(I), and Cr(III) in a detailed study, which found that Hg(II) complexes showed enhanced antibacterial activity relative to other complexes [33].

In our continuous research, we have successfully synthesized a novel 1D Hg(II) CP (**1**) as well as elucidated its crystal structure. The methodology employed for the preparation of single crystals, which includes 1D Hg(II) CPs (Hg‐MOCP), involved a straightforward layering deposition technique combined with sonochemistry. Furthermore, we assessed the antimicrobial properties of the synthesized materials against both Gram‐positive and Gram‐negative bacterial strains, in addition to investigating their anticancer potential. Also, the minimum inhibitory concentration (MIC) and minimum bactericidal concentration (MBC) values corroborate its bactericidal efficacy.

## 2. Experimental

### 2.1. Materials

The chemicals and reagents employed in this study were sourced from Sigma‐Aldrich and Merck, and they were utilized in their supplied form without the need for additional purification. Solutions were prepared using methanol obtained from Zagros Petrochemical.

### 2.2. Synthesis of Compound (**1**)

#### 2.2.1. Synthesis of [(μ_2_‐Cl)(Ina)Hg(μ_3_‐Cl)Hg(μ_2_‐Cl)_2_(Ina)]_n_ as Single Crystal (**1**)

A fabricated solution of isonicotinic acid (0.123 g, 1 mmol) in methanol (10 mL) was carefully layered onto the surface of a clear solution of HgCl_2_ (0.813 g, 3 mmol) in 10 mL of deionized water within a laboratory tube. The tube was then located in a stable environment. After a period of 1 week, colorless single crystals of (**1**) were obtained. The obtained products were collected and deemed suitable for X‐ray crystallography.

#### 2.2.2. Sonochemical Synthesis of [(μ_2_‐Cl)(Ina)Hg(μ_3_‐Cl)Hg(μ_2_‐Cl)_2_(Ina)]_n_ Nanostructure (**1**′)

To synthesize nanostructures of (**1**′) via the sonochemical bath method, a high‐density ultrasonic bath was directly immersed in a solution of isonicotinic acid prepared at a concentration of 0.01 M in 20 mL of methanol. Subsequently, 20 mL of 0.03 M aqueous solution of HgCl_2_ was added dropwise to the previous mixture. The solution was subjected to ultrasound irradiation at a temperature of 30°C for a duration of 60 min, resulting in the formation of a colorless powder identified as (**1**′). The resulting precipitates were filtered and air‐dried to yield the final product. IR (cm^−1^) selective bands: *ν* = 3440, 3012 (s), 1721, 1658, 1430, 1418, 644, and 522.

### 2.3. Characterization Instruments

Sonochemical experiments were performed using an Ultrasonic Bath Model vCLEAN. Fourier‐transform infrared (FT‐IR) analysis was performed with FT‐IR 4600 Type A spectrophotometers. Powder X‐ray diffraction (PXRD) measurements were carried out using a Philips X’pert diffractometer, employing monochromated MoKα radiation (*λ* = 0.71 Å). PXRD powder patterns were simulated with Mercury software based on single‐crystal X‐ray data. The analysis of crystal structure was conducted using ToposPro software, Version 5.5.2.0 [34]. TGA was performed using a Perkin Elmer STA 6000 apparatus, covering a temperature range from 50°C to 800°C. Additionally, scanning electron microscopy (SEM) characterization was conducted using a FESEM‐VEGA3 instrument. For cytotoxicity calculations, GraphPad Prism software was employed.

#### 2.3.1. X‐Ray Crystallography

Single‐crystal X‐ray diffraction studies were conducted on colorless crystals of compound (**1**) utilizing a Xcalibur four‐circle geometry diffractometer with an Atlas two‐dimensional area CCD detector at room temperature. The experiment employed MoKα radiation (*λ* = 0.71073 Å) in conjunction with a graphite monochromator. Data acquisition was executed using CrysAlis CCD software (Version 1.171.38.43, Rigaku Oxford Diffraction, 2015), while the refinement of the unit cell parameters was performed with the CrysAlis RED software package [35]. The determination of the crystal structure was achieved through direct methods using SHELXS2014/5, which effectively located the majority of nonhydrogen atom positions. Absorption corrections were implemented via spherical harmonics utilizing the SCALE3 ABSPACK algorithm. Subsequent refinement of the structure was carried out with SHELXL2018/3, applying anisotropic thermal displacement parameters to enhance precision. Molecular graphics for visualization purposes were produced using Brandenburg software. The final refinement process involved least‐squares fitting on *F* ^2^, resulting in an *R*‐factor of 0.0454 for reflections where *F* ^2^ exceeded 2*σ* (*F* ^2^). Molecular graphics for visualization purposes were produced using the Diamond 3.0 program [36].

### 2.4. Antimicrobial Activity Assay

#### 2.4.1. Disk Diffusion Method

This method, based on CLSI, M100‐S22, was used to assess the antibacterial activity of the synthesized nanoparticle (**1**′) against seven Gram‐positive and nine Gram‐negative bacterial strains at a concentration of 5 mg/mL in DMSO solution (Table 1). To test against certain pathogenic bacteria, sterile 6‐mm discs were impregnated with 25 μL of (**1**′) solution. Gram‐positive bacteria were controlled with penicillin discs (10 μg), whereas Gram‐negative bacteria were controlled with ciprofloxacin discs (5 μg). The negative control used was deionized water, and the lack of antibacterial effect of DMSO solvent on bacteria was also tested. A single colony was cultured in Brain Heart Infusion (BHI) broth (Merck, Germany) for an entire night, with the turbidity set to 0.5 McFarland standards, to create bacterial suspensions. Merck, Germany’s Mueller–Hinton Agar, or Mueller–Hinton Agar enhanced with 5% sheep blood (for *Streptococcus* spp.) was used for bacterial culture. Using sterile cotton swabs, the produced bacterial suspensions were equally distributed on the agar plates. The plates were then incubated for 24 h at 37°C either in a candle jar (for *Streptococcus* spp.) or under ambient conditions. Every experiment was carried out in duplicate, and the diameter of the no‐growth halo around the well was measured using a ruler [37].

**TABLE 1:** Clinical and standard bacteria lists used in this study.

Gram‐positive bacteria	Gram‐negative bacteria
*Staphylococcus aureus* ATCC6538	*Pseudomonas aeruginosa* ATCC 15442

*Staphylococcus epidermidis* ATCC14990	*Klebsiella pneumoniae* ATCC13883

Methicillin‐resistant *Staphylococcus aureus* (MRSA) clinical	*Pseudomonas aeruginosa* clinical (PAO1)

*Enterococcus faecalis* ATCC1394	*Vibrio cholerae* clinical NAG

*Streptococcus pyogenes* ATCC19615	*Proteus mirabilis* ATCC4371

*Streptococcus agalactiae* PTCC1768	*Escherichia coli* ATCC8739

*Bacillus cereus* ATCC11778	*Shigella flexeneri* ATCC12022
	*Acinetobacter baumannii* ATCC 19606
	*Salmonella thyphi* ATCC14029 PTCC1609

#### 2.4.2. Broth Microdilution Method

The standard broth microdilution method, CLSI, M100‐S22, was used to determine the MIC and MBC of (**1**′) nanoparticle for the bacterial strains that demonstrated growth inhibition in the disk diffusion assay. Bacterial cultures were cultivated overnight at 37°C in Mueller–Hinton Broth (Merck, Germany) and adjusted to a 0.5 McFarland standard in fresh, sterile Mueller–Hinton broth to calculate the MIC. A 96‐well microtiter plate was then filled with 100 μL of Mueller–Hinton broth in each well. 100 μL of each bacterial solution (diluted 1:150 in 0.9% saline) was introduced to each well after (**1**′) nanoparticle at a concentration of 5 mg/mL was serially diluted throughout the wells. The microplates were incubated at 37°C for 24 h. Each test was conducted twice [38, 39].

### 2.5. MTT Assay

#### 2.5.1. Cell Culture

Two distinct cell lines, VERO (normal) and OVCAR3 (cancer), were sourced from Iranian Research Institutions: The Iranian Biological Resource Center and the Stem Cell Biology Research Center, both located in Yazd. Standard cell culture techniques were employed to maintain these lines. Specifically, the cells were cultured in T25 flasks, a standard vessel for *in vitro* cell propagation. A complete growth medium, essential for cell survival and proliferation, was utilized. This medium was based on Dulbecco’s modified eagle medium (DMEM), a widely used basal medium providing essential nutrients. To this, several supplements were added: 10% fetal bovine serum (FBS), a rich source of growth factors crucial for cell division and function; 1% sodium bicarbonate, acting as a buffering agent to maintain the physiological pH necessary for cell viability; and 1% Penicillin–Streptomycin (Pen–Strep), a combination antibiotic used to prevent contamination by bacteria and fungi, thereby ensuring the integrity of the cell cultures. The culture medium was regularly replenished every two to three days to provide a consistent supply of nutrients and remove metabolic waste products, thus promoting optimal cell growth and maintaining cell health.

Following near‐complete confluence (approximately 80%) in 25 cm^2^ flasks, cells were passaged. The spent medium was removed, and cells were rinsed with 1× PBS. A 0.025% Trypsin‐EDTA solution was used to detach cells, followed by a 3‐min incubation at 37°C. Trypsin activity was then neutralized by adding complete culture medium. The resulting cell suspension was centrifuged (3000 rpm, 5 min), the supernatant discarded, and the cell pellet resuspended in fresh medium. The 1 × 10^4^ cells were seeded into each well of a 96‐well plate and cultured in DMEM with 10% FBS for 24 h for recovery. Subsequently, the medium was replaced with fresh DMEM containing 10% FBS and varying concentrations (0.98–1000 μg/mL) of (**1**′) nanoparticles. After a 3‐day incubation, cell viability was assessed using an MTT assay. Following a 4‐h incubation with MTT, the resulting formazan crystals were dissolved in DMSO, and absorbance was measured at 570 nm using an ELISA reader. Absorbance values correlated with viable cell number [40].

#### 2.5.2. Statistical Analysis

Statistical analysis of the experimental data was carried out using IBM SPSS Statistics 22 and GraphPad Prism (Version 7.0a), two commonly used software packages for statistical computing and data visualization. A one‐way ANOVA was performed to compare the means of the control and experimental groups, determining whether any statistically significant differences existed among them. To pinpoint specific differences between individual groups, a post hoc Tukey test was applied. The results of these analyses are presented as mean values with their corresponding standard errors (mean ± SEM). A *p* value of less than 0.05 was adopted as the criterion for statistical significance, meaning that differences observed between groups with *p* < 0.05 were considered unlikely to be due to chance.

## 3. Results and Discussion

### 3.1. Synthetic Perspective

**FIGURE 1:**
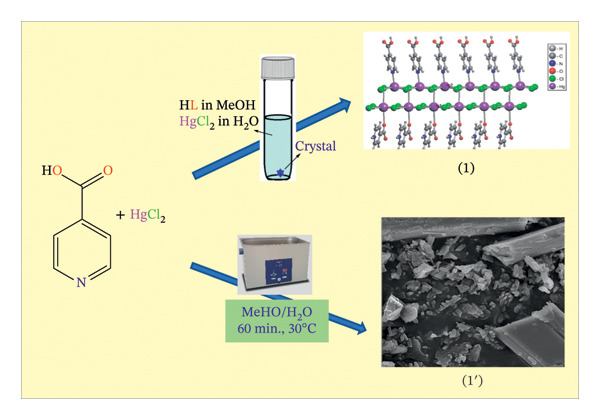
The synthesized methods of compound as a single crystal (**1**) and nanostructures (**1**′).

Compound (**1**) was synthesized in situ by reacting 4‐pyridinecarboxylic acid and HgCl_2_ in a 1:3 stoichiometric ratio in water and methanol, while compound **1**′ was obtained by reacting o 4‐pyridinecarboxylic acid with HgCl_2_ in a 1:3 stoichiometric ratio in water and methanol by ultrasonic assistance as shown in Figure 1.

### 3.2. X‐Ray Single Crystal Structure

#### 3.2.1. Crystal Structure Description

**FIGURE 2:**
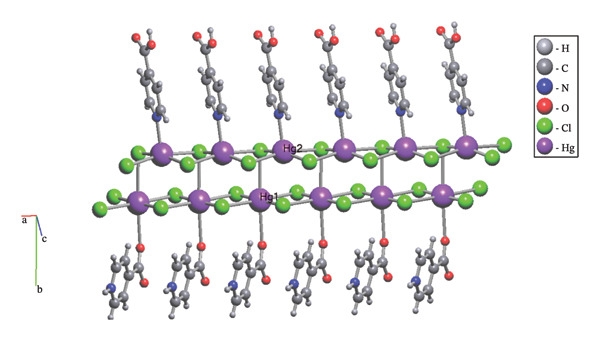
X‐ray molecular structures show a segment of the 1D framework present in compound (**1**).

The structure of (**1**) was confirmed using single‐crystal X‐ray diffraction to determine its solid‐state arrangement. Compound (**1**) is classified as a 1D CP and features a monoclinic crystal system with the space group Pc (Table 2). In the symmetric unit, compound (**1**) comprises two mercury ions and two isonicotinic acid ligands, as illustrated in Figure 2. This compound features two mercury atoms exhibiting distinct coordination numbers, along with two isonicotinic ligands that coordinate differently: one ligand interacts through the nitrogen atom, while the other engages through the oxygen atom.

**TABLE 2:** Crystal data and structures refinement for compound (**1**).

Crystal data	Compound (**1**)
Chemical formula	C_6_H_5_Cl_2_HgNO_2_·C_6_H_5_NO_2_·Cl_2_Hg
*M*_*r*_	789.20
Crystal system, space group	Monoclinic, Pc
Temperature (K)	295
*a*, *b*, *c* (Å)	3.9045 (2), 18.5529 (12), 12.8519
*β* (°)	92.087 (5)
*V* (Å^3^)	930.37 (10)
*Z*	2
Radiation type	Mo Kα
*µ* (mm^−1^)	17.074
Crystal size (mm)	0.28 × 0.12 × 0.08
Diffractometer	Xcalibur, atlas
Absorption correction	Multiscan
*T*_min_, *T*_max_	0.7912, 1.0000
No. of measured refls	13815
No. of independent refls	4419
No. of observed refls [*I* > 2*σ* (*I*)]	3105
*R*_int_	0.0617
(sin *θ*/*λ*)_max_ (Å^−1^)	0.692
*R*[*F* ^2^ > 2*σ* (*F* ^2^)], *w* *R* (*F* ^2^), S	0.0454, 0.0593, 1.03
No. of reflections	4419
No. of parameters	218
Flack parameter	−0.027 (10)
H‐atom treatment	H‐atom parameters constrained
Δ*ρ*_max_, Δ*ρ*_min_ (e Å^−3^)	+0.827, −0.850
CCDC	2354283

Hg1 is coordinated by one oxygen atom from isonicotinic acid and one chlorine atom, which forms a triple bridge, while four additional chlorine atoms are arranged in a double bridge configuration. The bond lengths are measured as follows: Hg1‐O1 at 2.857(4), Hg1‐Cl1 at 2.299(5), Hg1‐Cl2 at 2.312(5), and Hg1‐Cl3^*i*^ at 3.096(5) Å. The angles surrounding the Hg1 center are recorded as Cl1‐Hg1‐O1 at 90.2(3)°, 91.6(3), Cl2‐Hg1‐Cl3^*i*^ at 88.03(4)°, and Cl1‐Hg1‐Cl3^*i*^ at 90.3(3)°. These findings suggest a twisted octahedral geometry for the structure, with Hg1 exhibiting a coordination number of six (Figure 3(a)). The Hg2 atom is coordinated to four chlorine atoms, with two forming double bridges, two forming triple bridges, and one nitrogen atom from the isonicotinic acid ligand. These results show a coordination number of five for Hg2, with bond lengths of [Hg2‐Cl4 = 2.357(5), Hg2–Cl3 = 2.373(5), Hg2‐N1 = 2.407(14), Hg2‐Cl4^*ii*^ = 2.963(6) Å]. The angles surrounding Hg2 are measured as follows: N1‐Hg2‐Cl3 = 96.1(4)°, Cl3‐Hg2‐Cl4 = 90.4(2)°, and Cl4‐Hg2‐Cl4^*ii*^ = 93.8(2)° (Table 3). Consequently, a square‐based pyramidal geometry is suggested for this molecular structure (Figure 3(b)).

FIGURE 3The coordination environment around of (a) Hg1 and (b) Hg2.

**Figure:**
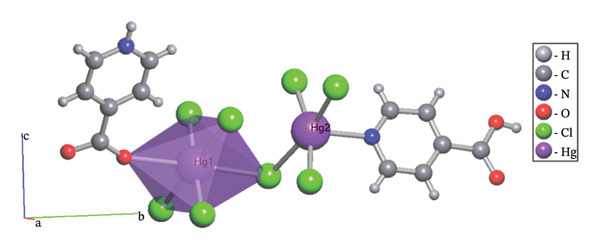
(a)

**Figure:**
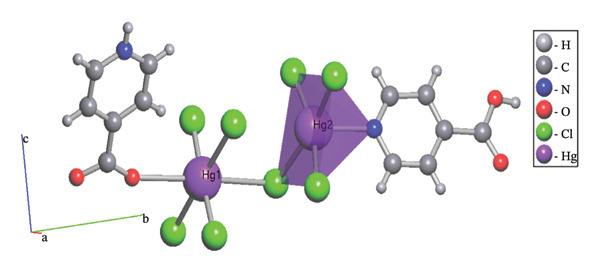
(b)

**TABLE 3:** The selected bond lengths (Å) and angles (°) for compound (**1**).

Bond lengths (Å)		Bond angles (°)	
Hg1‐Cl1	2.298 (5)	Cl1‐Hg1‐O1	90.2 (3)
Hg1‐Cl2	2.312 (5)	Cl2‐Hg1‐Cl3^*i*^	88.0 (4)
Hg1‐O1	2.857 (4)	Cl1‐Hg1‐Cl3^*i*^	90.3 (3)
Hg1‐Cl3^*i*^	3.096 (5)	Cl1‐Hg2‐Cl1^*i*^	91.5 (3)
Hg2‐N1	2.407 (14)	Cl3‐Hg2‐Cl4^*i*^	90.4 (3)
Hg2‐Cl4	2.357 (5)	N1‐Hg2‐Cl3	96.1 (4)
Hg2‐Cl3	2.373 (5)	N1‐Hg2‐Cl4	88.1 (5)
Hg2‐Cl4^*ii*^	2.963 (6)	Cl4‐Hg2‐Cl4ʹ	93.8 (2)

*Note:* Symmetry codes: (i) *x* − 1, *y*, *z*; (ii) *x* + 1, *y*, *z*.

A brief review of similar compounds of mercury ion has been conducted, which usually show coordination numbers 4 and 5 and tetrahedral and square pyramidal geometrical arrangements, respectively [29, 31, 41, 42]. However, compound (**1**) is a polymer with two mercury ions, which have 5 and 6 coordination with square pyramidal and octahedral geometrical arrangements. The results of the comparison of some selected compounds with compound (**1**) are shown in Table 4.

**TABLE 4:** The structural data of compound (**1**) compared with the reported works.

Compound	Geometric structure	C.N	[Ref]
{[Hg(L)(Cl)_2_]}_*n*_·(ACN)_0.341_L = dispiro–dipyridyloxy–cyclotriphosphazene	Distorted tetrahedral	4	[29]

[HgCl_2_(2,3‐pydc)](2‐ampyH)_2_·2H_2_O	Square pyramidal	5	[31]

[HgCl(2,6‐pydc)(H_2_O)](4‐ampyH)	Square pyramidal	5	[31]

{[HgCl(pic)]}_n_·pic = 2‐pyridinecarboxylic acid	Distorted tetrahedral	4	[41]

[HgCl(pic)(picH)]	Square pyramidal	5	[41]

[Hg(L)(SCN)_2_]·*L*′…*L* = 2‐pyridinecarboxylic acid and *L*′ = 2‐amino‐3‐methylpyridine	Square pyramidal	5	[42]

Compound (**1**)	Twisted octahedral (Hg1)	6	This work
	Square pyramidal (Hg2)	5	

#### 3.2.2. Supramolecular Chemistry

The study examined various intermolecular interactions, including hydrogen bonds, Van der Waals forces, and intramolecular covalent bonds, within (**1**). The intermolecular hydrogen bonds in the structure are formed between the oxygen atoms associated with the carboxylic acid group of the isonicotinic acid ligand and the hydrogen atoms of both the carboxylic acid group and the nitrogen atom of the same ligand (Figure 4(a)). Distances corresponding to the established hydrogen bonds demonstrate the appropriate measurements necessary for the interactions. Also, van der Waals interactions in compound (**1**) are shown in Figure 4(b).

FIGURE 4(a) Hydrogen bonds and (b) Van der Waals interactions in compound (**1**).

**Figure:**
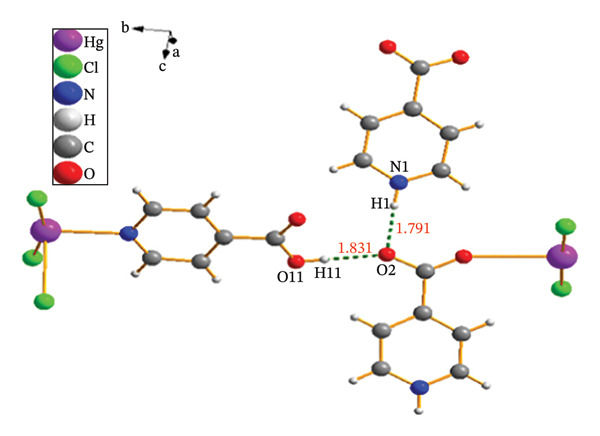
(a)

**Figure:**
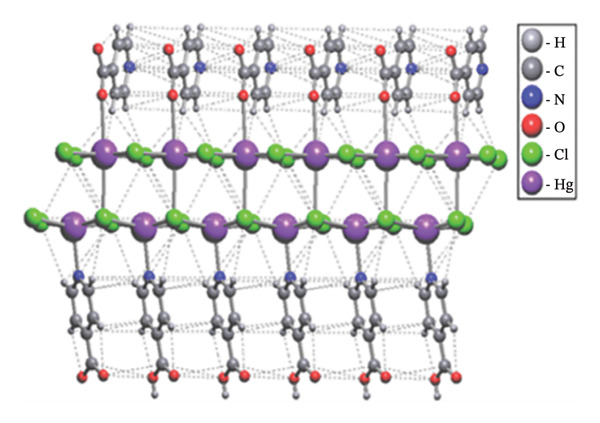
(b)

A further interaction contributing to the ultimate configuration of compound (**1**) is the π–π interaction present between the aromatic rings, as illustrated in Figure 5(a). Figure 5(b) depicts this π–π interaction along with the distance separating the aromatic rings within the structure of compound (**1**). Research indicates that the optimal distance for establishing π–π interactions ranges from 3.904 to 3.905 Å.

FIGURE 5(a) π–π interactions between pyridine rings. (b) The distance between aromatic rings in compound (**1**).

**Figure:**
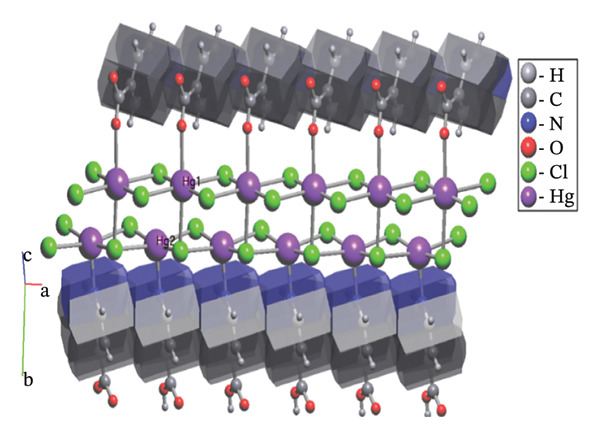
(a)

**Figure:**
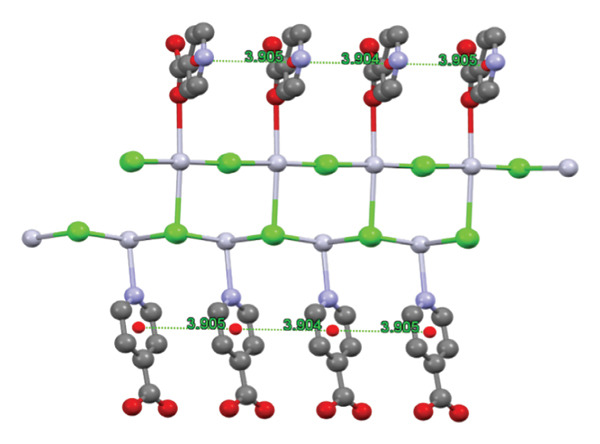
(b)

**FIGURE 6:**
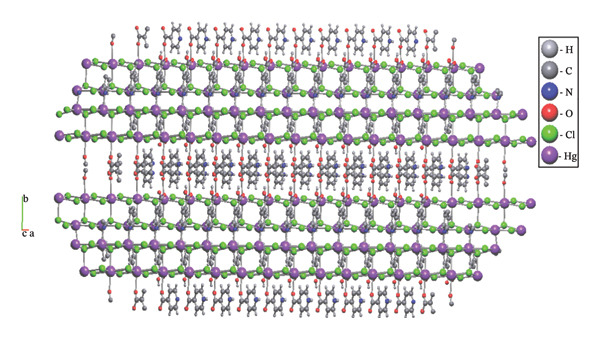
3D crystal packing of compound (**1**).

An overview of the three‐dimensional structure of polymer (**1**) is shown in Figure 6.

#### 3.2.3. Analysis of Topology

**FIGURE 7:**
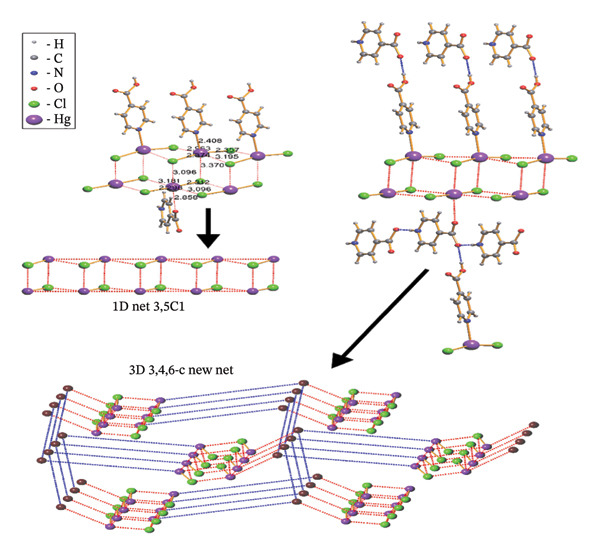
The hierarchy of interactions and underlying topologies for compound (**1**).

The structure of (**1**) is constructed in a hierarchical manner with interactions of varied strength (Figure 7). The strongest coordination interactions of the length 2.298–2.408 Å form molecular (0D) complexes [HgCl_2_] and [HgCl_2_(Ina)]. The complexes are further connected to each other and the zwitterion 4‐carboxypyridinium through the set of weaker interactions of the length 2.858–3.370 Å to form 1D CP of the underlying topology 3,5C1, as can be found by ToposPro software [34, 43]. The 1D polymeric group contacts with four of the same polymeric groups by H‐bonds to form 3D supramolecular framework with the underlying topology of a new type (point symbol for the 3,4,6‐c net is {3.4^2^}_2_{3^2^.4^2^.5^3^.6^4^.7^3^.9}_2_{6.8^4^.10}) [44–47].

#### 3.2.4. Hirshfeld Surface Analysis

The investigation focused on the intermolecular interactions as well as crystal structures of (**1**), employing Hirshfeld surface analysis alongside 2D fingerprint diagrams, facilitated by the Crystal Explorer 3.0 software. The 3D *d*_norm_ surface proved effective for visualizing these interactions. This method utilizes normalized distance functions, denoted as *d*_*e*_ and *d*_*i*_, which represent the distances from a specific point on the surface to the nearest external as well as internal atoms, respectively. In the *d*_norm_ Hirshfeld surface, the colors blue, white, and red signify interatomic contacts that are longer than, equal to, or shorter than the standard van der Waals distances, as illustrated in Figure 8, which presents the Hirshfeld analysis surface diagram of (**1**) in three distinct states: *d*_norm_, *d*_*i*_, and *d*_*e*_ [48–50]. Figure 9 shows the contacts of various bonds from the Hirshfeld surface in the structure of (**1**). The distances d_norm_, *d*_*i*_, and *d*_*e*_ were calculated through the software and are shown in Table 5.

FIGURE 8HSA of compound (**1**): (a) normalized distance (*d*_norm_); (b) distance from a point on the Hirshfeld surface to the nearest internal nucleus (*d*_*i*_); and (c) distance from a point on the Hirshfeld surface to the nearest external nucleus (*d*_*e*_).

**Figure:**
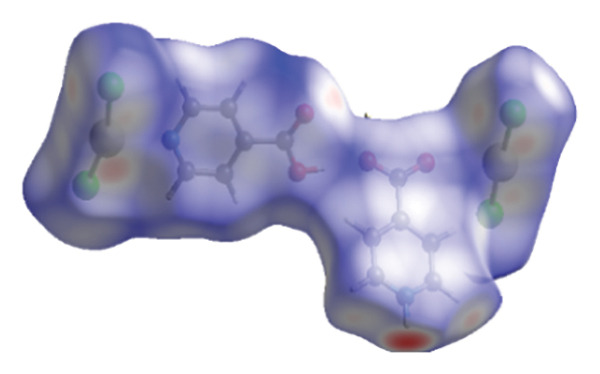
(a)

**Figure:**
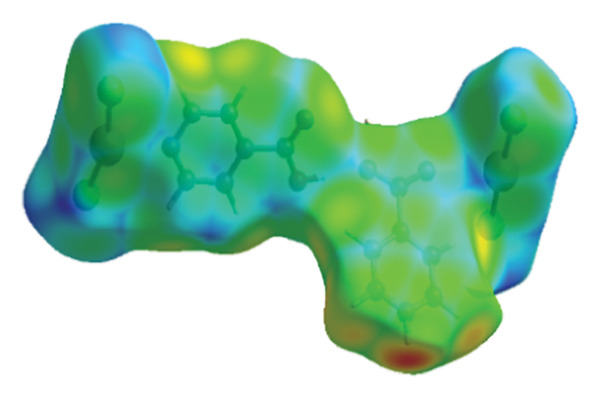
(b)

**Figure:**
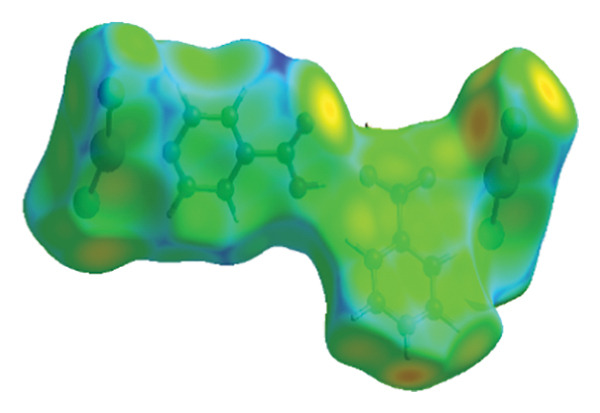
(c)

FIGURE 9(a) Close contacts (O–H), (b) equidistant contacts (C–H), and (c) long‐distance contacts (C–C) from the Hirshfeld surface in the structure of compound (**1**).

**Figure:**
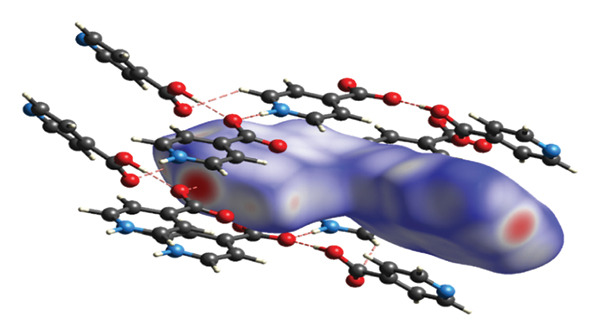
(a)

**Figure:**
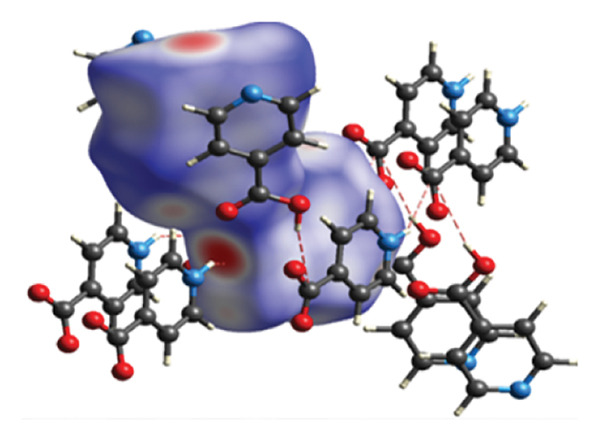
(b)

**Figure:**
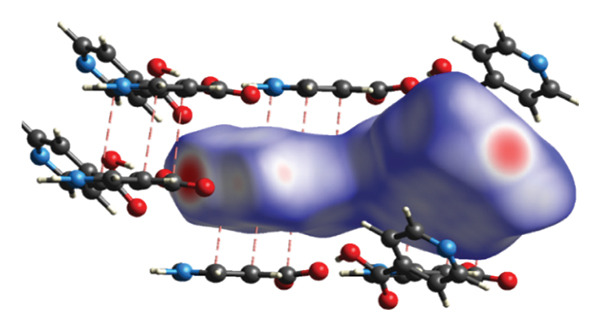
(c)

**TABLE 5:** *d*_norm_, *d*_*i*_, and *d*_*e*_ distances calculated through crystal explorer software.

Property	Units	Min	Max	Mean
*d*_norm_	Å	−0.7517	1.2980	0.4157
*d*_*i*_	Å	0.6422	2.4486	1.6712
*d*_*e*_	Å	0.6412	2.8170	1.9005

*Note:* The negative sign suggests that the distances from the Hirshfeld surface are very close.

**FIGURE 10:**
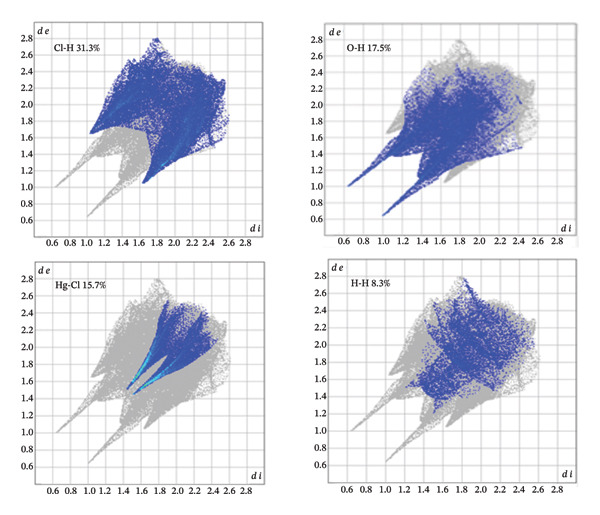
Fingerprint plots of major contacts in compound (**1**).

Using fingerprint diagrams from Hirshfeld analysis and Crystal Explorer software (Figure 10) illustrates the role of intermolecular interactions (hydrogen bonds and various van der Waals interactions) in (**1**). The fingerprint diagrams’ blue regions show how much the measured interaction contributed to the compound’s structure, and the gray areas represent the entire surface.

**FIGURE 11:**
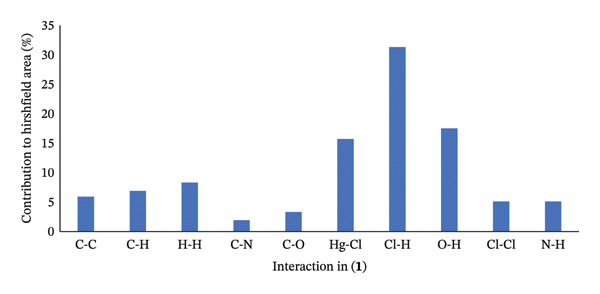
The relative contribution intermolecular to the Hirshfeld surface area for compound (**1**).

In compound (**1**), the primary interaction is attributed to Cl…H/H…Cl, which constitutes 31.3% of the overall Hirshfeld surface. Other significant interactions include O…H at 17.5%, Hg…Cl at 15.7%, and H…H at 8.3%, which collectively represent the most substantial contributions to the Hirshfeld surface. The lattice stability is reinforced by both hydrogen bonds and dispersion forces. The relative contributions of intermolecular interactions in compound (**1**) are depicted in Figure 11.

This compound is characterized as a 1D CP of mercury, exhibiting 5 and 6 coordination within a monoclinic crystal system classified under the Pc space group, as determined through comprehensive analysis and research. The geometric configurations suggested for this compound include octahedral and twisted square pyramidal shapes, inferred from the bond angles and lengths surrounding the central atom. A thorough examination of the types of interactions present in this structure revealed that the predominant noncovalent intermolecular forces facilitating the formation of this CP are hydrogen bonds (H…Cl and H…O) and π–π interactions between the aromatic rings of two molecules. Additionally, other bonding interactions forming the aromatic rings, such as C…C and C…N, were also identified.

### 3.3. Characterization of Compound (**1**′)

#### 3.3.1. IR Spectra

**FIGURE 12:**
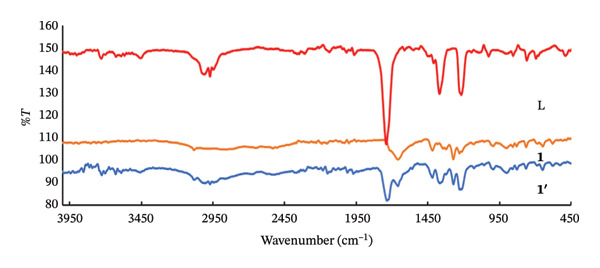
FT‐IR spectra of the ligand (L) and compounds (**1**) and (**1**′).

By examining the FT‐IR spectrum of the ligand isonicotinic acid (**L**), the absorption band related to H–O in the ligand is seen at a frequency of 3458 cm^−1^, which shifted to 3440 cm^−1^ in (**1**′) (Figure 12). In the FT‐IR spectrum of isonicotinic acid (L), the absorption band related to the H‐C group is at a frequency of 3010–2966 cm^−1^, which is observed around 3012 cm^−1^ in nanoparticles (**1**′) [51]. The absorption band corresponding to the O=C stretching frequency of the isonicotinic acid ligand is observed at 1745 cm^−1^ and shifted to 1721 cm^−1^ in (**1**′), which also clearly indicates the coordination of the isonicotinic ligand to the mercury ion. Two absorption bands corresponding to the uncoordinated O=C asymmetric and symmetric stretching frequency are observed, around 1658 and 1418 cm^−1^ in the FT‐IR spectrum of the nanoparticle (**1**′). The difference in the stretching frequencies of the symmetric and asymmetric CO is 214 cm^−1^ in (**1**′). While the difference in the stretching frequencies of CO in this ligand in the free state is 200 cm^−1^ and the greater this difference than in the free state indicates that the position of the ligand (L) in the compound (**1**′) is monodentate [52, 53]. The absorption peak at 1430 cm^−1^ represents the stretching vibration of C=C bonds in the ligand. The presence of weak peaks at 644 and 522 cm^−1^ indicates the stretching vibrations of the O–Hg and N–Hg bonds.

#### 3.3.2. TGAs

**FIGURE 13:**
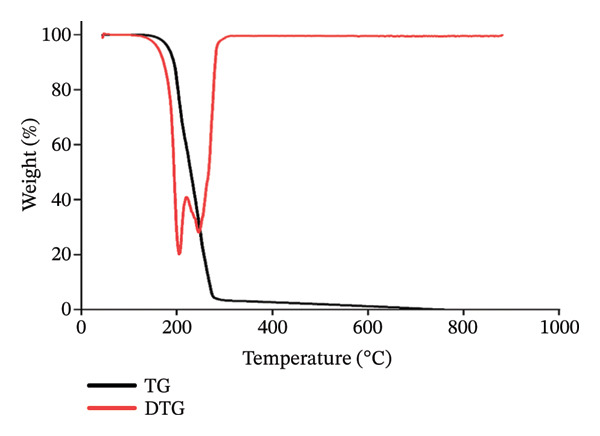
TG‐DTG diagrams of (**1**′).

The thermal stability of the compound was evaluated between 50°C and 800°C under nitrogen gas. TGA was performed on a sample of (**1**′) at a heating rate of 10°C/min. Figure 13 shows the TGA and its derivative (DTG) diagrams for nanoparticles (**1**′). As can be seen, the TGA plot shows a two‐step weight loss of (**1**′), with the entire (**1**′) being removed in two closely spaced steps, starting at 160°C and ending at 400°C. This is a characteristic of mercury compounds that can be removed all at once in a thermal calorimetric analysis [29].

#### 3.3.3. X‐Ray Powder Diffraction

**FIGURE 14:**
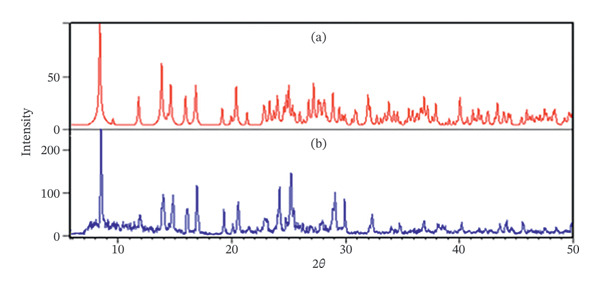
The XRD patterns of (a) simulated from single‐crystal X‐ray data of compound (**1**) and (b) nanoparticle of (**1**′).

X‐ray diffraction serves as a robust analytical technique for investigating the structural characteristics of materials, particularly inorganic and organometallic compounds. A key application of this method is to ascertain the nanoparticle form of a material. Researchers achieve this by comparing the XRD diffraction patterns of both crystal and nanoparticle samples. A congruence in these patterns suggests that the molecular architecture of the nanoparticles, synthesized via the sonochemical approach, closely resembles that of a single crystal. Figure 14 presents the diffraction patterns for both the nanoparticles and the single crystal. The analysis, supported by prior research, indicates a significant correlation between the diffraction bands, thereby affirming that the crystal and nanoparticles exhibit analogous structural properties.

#### 3.3.4. SEM Analysis

FESEM analysis is one of the key techniques used to study the surface structure of materials at both the micrometer as well as nanometer scales. Unlike optical microscopes, SEMs offer an unmatched ability to closely examine material surfaces. Figure 15(a) shows FESEM images of (**1**′), which reveal that the polymer’s structure is likely to be rod‐shaped based on its appearance. The size of nanoparticle (**1**′) was calculated using the Image J program and the particle size frequency chart is shown in Figure 15(b). According to the chart, the highest frequency of particle sizes is between 90 and 100 nm.

FIGURE 15(a) SEM image and (b) corresponding diameter size distribution histogram of compound (**1**′).

**Figure:**
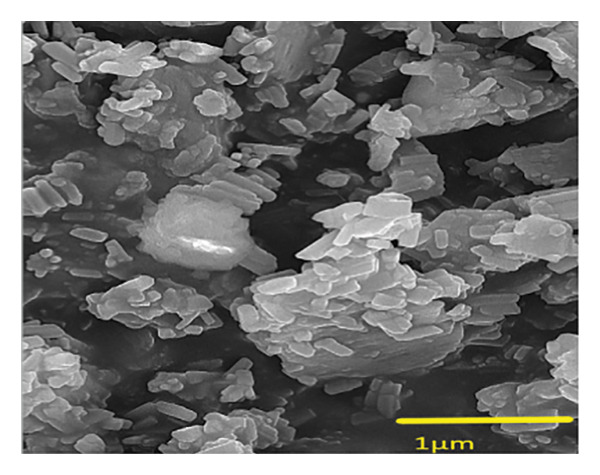
(a)

**Figure:**
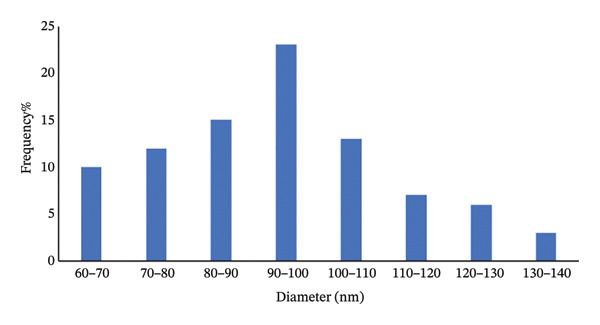
(b)

### 3.4. Biological Studies

The antibacterial properties of nanoparticles (**1**′) were assessed using the disk diffusion method and the broth microdilution assay. The diameters of the inhibition zones (mm) along with the MIC and MBC values (μg/mL) were determined for a variety of Gram‐positive and Gram‐negative bacterial strains.

#### 3.4.1. Disk Diffusion Assay

**FIGURE 17:**
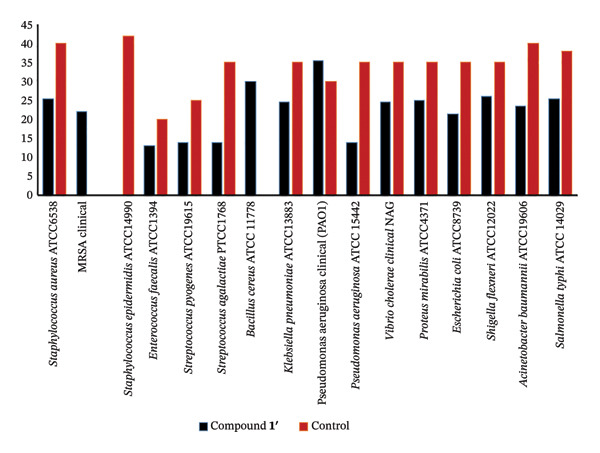
Antibacterial activity of nanoparticle (**1**′) against the bacteria. The size of the inhibition zones around the disk loaded the nanoparticle is shown in the vertical axis (mm). The inhibition zones of (**1**′) against bacteria are presented on the left side of the table, while the right side of the figure illustrates the inhibition zones of the control antibiotics, penicillin and ciprofloxacin, against Gram‐positive and Gram‐negative bacteria, respectively.

The findings from the disk diffusion assay indicated that nanoparticle (**1**′) showed considerable antibacterial effectiveness against both Gram‐positive and Gram‐negative bacteria. The most significant inhibition zone was found for *Pseudomonas aeruginosa* (PAO1), measuring 35.5 mm, followed by *Bacillus cereus* (30 mm), *Klebsiella pneumoniae* (24.5 mm), and *Staphylococcus aureus* (ATCC6538, 25.5 mm). Among the Gram‐positive bacteria, *Streptococcus pyogenes* and *Streptococcus agalactiae* showed moderate sensitivity with inhibition zones of 14 mm. The most minor inhibition zones were noted for *Enterococcus faecalis* (13 mm) and *Staphylococcus epidermidis* (0 mm) that was resistant to the nanoparticle (Figures 16 and 17).

FIGURE 16Antibacterial activity assay of (**1**′) by the disk diffusion method. (a) The inhibition zone produced by *Staphylococcus aureus*, when exposed to the control group. (b) The inhibitory zone observed for *S. aureus*. (c) The inhibition zone generated by methicillin‐resistant *Staphylococcus aureus* (MRSA) clinical isolate upon treatment with the nanoparticles (5 mg/mL). (d) The antibacterial effect demonstrated by *Vibrio cholerae* clinical NAG in response to (**1**′) (5 mg/mL).

**Figure:**
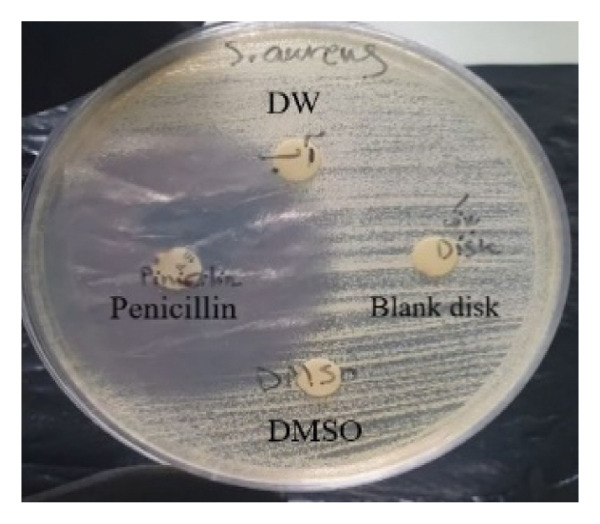
(a)

**Figure:**
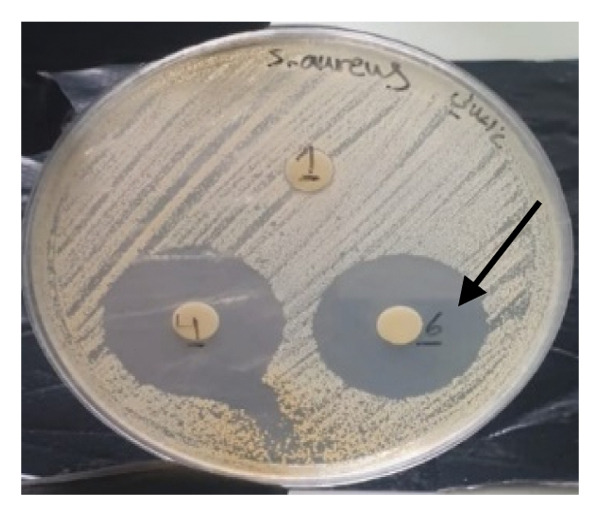
(b)

**Figure:**
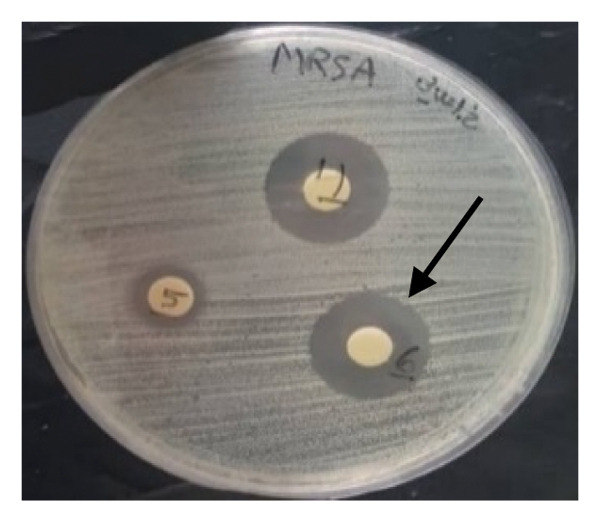
(c)

**Figure:**
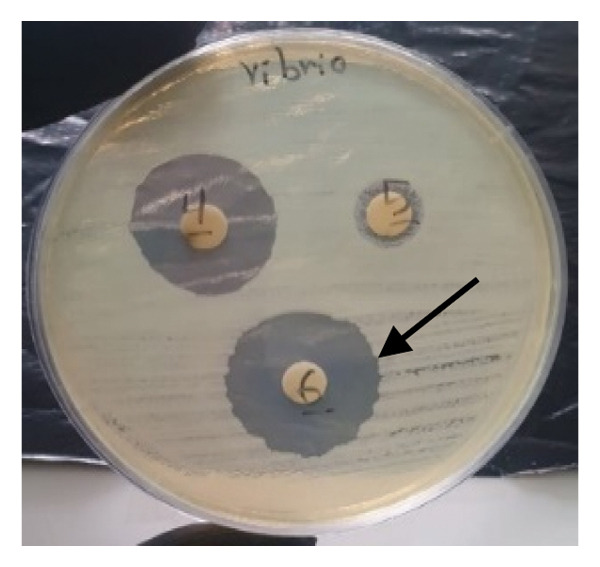
(d)

#### 3.4.2. Broth Microdilution Assay

The MIC and MBC values further validated the effectiveness of the nanoparticle against different bacterial strains. The lowest MIC values (0.12 μg/mL) were found for *Streptococcus pyogenes* and *Streptococcus agalactiae*, while *Proteus mirabilis* had the highest MIC (1250 μg/mL), indicating strong resistance. *Staphylococcus aureus*, *Escherichia coli*, *Klebsiella pneumoniae*, *Vibrio cholerae*, and *Salmonella typhi* displayed MIC values of 0.97 μg/mL, showcasing the intense antibacterial action of the nanoparticle. The MBC values were generally consistent with the MIC values, highlighting the bactericidal characteristics of the nanoparticle against most tested bacteria (Table 6).

**TABLE 6:** Antibacterial assay of nanoparticle (**1**′) against the bacteria by disk diffusion and broth microdilution methods.

Bacteria	Disk diffusion method		Broth microdilution method	
	Control^1^ (mm)^2^	Nanoparticle (mm)	MIC nanoparticle (μg/mL)	MBC nanoparticle (μg/mL)
*Staphylococcus aureus* ATCC6538	40	25.5	0.97	0.97
MRSA^3^ clinical	0	22	4.88	4.88
*Enterococcus faecalis* ATCC1394	20	13	0.24	0.97
*Streptococcus pyogenes* ATCC19615	25	14	0.12	0.12
*Streptococcus agalactiae* PTCC1768	35	14	0.12	0.12
*Bacillus cereus* ATCC 11778	0	30	0.48	0.48
*Klebsiella pneumoniae* ATCC13883	35	24.5	0.97	0.97
*Pseudomonas aeruginosa* clinical (PAO1)	30	35.5	1.95	1.95
*Pseudomonas aeruginosa* ATCC 15442	35	14	9.76	9.76
*Vibrio cholerae* clinical NAG	35	24.5	0.97	0.97
*Proteus mirabilis* ATCC4371	35	25	1250	1250
*Escherichia coli* ATCC8739	35	21.5	0.97	0.97
*Shigella flexneri* ATCC12022	35	26	0.48	0.48
*Acinetobacter baumannii* ATCC19606	40	23.5	1.95	1.95
*Salmonella typhi* ATCC 14029	38	25.5	0.97	0.97

^1^Penicillin (10 μg) for Gram‐positive or ciprofloxacin (5 μg) for Gram‐negative were used as a positive control and DMSO and DDW were used as a negative control in the disk diffusion method.

^2^Millimeter.

^3^Methicillin‐resistant *Staphylococcus aureus* (MRSA).

In this investigation, MIC and MBC values of the CP were assessed to analyze its antibacterial properties [54]. The variations in MIC and MBC values across different bacterial strains can be explained by differences in cell wall composition, membrane permeability, and interactions at the molecular level, including hydrogen bonding and π–π stacking [54, 55]. According to established guidelines, a ratio of MBC/MIC ≤ 4 suggests bactericidal activity, while a ratio greater than 4 indicates bacteriostatic activity [55, 56]. These findings enhance the understanding of the distinctions between the compound’s bactericidal and bacteriostatic effects [56]. According to the results shown in Table 6, the MBC/MIC values for (**1**′) are less than 4, and, therefore, the compound shows strong bactericidal activity.

#### 3.4.3. Comparison With Standard Antibiotics

The antibacterial effectiveness of (**1**′) was compared with standard antibiotics. Penicillin was used as a positive control for Gram‐positive bacteria, while ciprofloxacin was used for Gram‐negative bacteria. In several instances, the nanoparticle showed comparable or superior inhibition zones relative to the standard antibiotics, especially against *Pseudomonas aeruginosa* (PAO1), *Bacillus cereus*, and *Shigella flexneri*.

Table 7 shows a comparison of MIC values of the ligand, the similar mercury compound and (**1**′). The MIC values for (**1**′) are lower than others and so nanoparticle (**1**′) shows better performance.

**TABLE 7:** MIC data of compound (**1**′) in comparison with the previous works.

Compounds	Bacteria	MIC	[Ref.]
Ina	*Escherichia coli*	> 2	[57]
	*Staphylococcus aureus*	> 2	

[Hg(PicA)(N_3_)]_*n*_	*Escherichia coli*	5000	[58]
	*Staphylococcus aureus*	1250	

Compound (**1**′)	*Escherichia coli*	0.97	This work
	*Staphylococcus aureus*	0.97	

The findings of this research demonstrate that the nanoparticle (**1**′) displayed significant antibacterial properties against both types of bacteria, Gram‐positive and Gram‐negative. However, it was generally more effective against Gram‐negative bacteria. Specifically, *Pseudomonas aeruginosa* (clinical PAO1) exhibited the largest inhibition zone (35.5 mm), followed by *Shigella flexneri* (26 mm) and *Klebsiella pneumoniae* (24.5 mm). In comparison, among the Gram‐positive bacteria, *Staphylococcus aureus* (ATCC6538) demonstrated a notable inhibition zone of 25.5 mm, while *Streptococcus pyogenes* and *Streptococcus agalactiae* each had moderate inhibition zones of 14 mm. The enhanced effectiveness against Gram‐negative bacteria may be due to the nanoparticle’s capability to penetrate the outer membrane, which is often a barrier to traditional antibiotics [59, 60].

Insights were enhanced through the broth microdilution technique. The MIC and MBC values for most Gram‐negative strains were consistently low (typically around 0.97 μg/mL), reinforcing the idea of strong bactericidal activity. However, regarding Gram‐positive bacteria, while some strains like *S. aureus* (ATCC6538) displayed comparably low MIC values, methicillin‐resistant *Staphylococcus aureus* (MRSA) required a higher concentration (4.88 μg/mL) to hinder growth. This difference may be linked to the innate resistance mechanisms and structural differences within the MRSA cell wall, which could impede the nanoparticle’s ability to reach intracellular targets.

A particularly significant finding was the effect of the nanoparticle on MRSA, a critical multidrug‐resistant pathogen. MRSA exhibited an inhibition zone of 22 mm, with the associated MIC and MBC values (4.88 μg/mL) indicating notable bactericidal effectiveness. Given that MRSA infections present a major global health threat due to their resistance to β‐lactam antibiotics, the nanoparticle’s potential to reduce MRSA growth is highly encouraging. This observation is consistent with the results of Malarvizhi et al, who showed that mercury‐based formulations can effectively target multidrug‐resistant bacterial strains while addressing toxicity concerns simultaneously. Despite the safety issues associated with mercury‐based compounds, their strong effectiveness against resistant strains like MRSA suggests promise if toxicity can be controlled or reduced through improved formulations [61].

The reported antimicrobial effectiveness of (**1**′) is further supported by previous research on organomercury compounds. For example, studies have shown that organomercury complexes with piperidinethiohydrazide ligand display significant antimicrobial properties, implying that integrating mercury into organic frameworks can enhance antibacterial action [62]. Our results are also consistent with those noted by Almasi et al. [57], who discovered substantial antibacterial properties in Ag(I) and Zn(II) isonicotinate complexes. Moreover, while Almási et al. investigated DNA interactions as a potential mechanism of action, our study mainly focused on the inhibition of bacterial growth. It is crucial to understand that bacterial strain susceptibility to nanoparticles can vary based on various physicochemical factors, such as solubility, morphology, size, surface charge, and the capacity to release biocidal metal ions, along with the inherent characteristics of the specific bacterial species [63].

Factors such as rapid bacterial growth, biofilm formation [63, 64], enzymatic detoxification mechanisms, volatilization processes, efflux pump activities, and genetic alterations significantly impact overall susceptibility to nanoparticles [38, 65]. The observed inhibition zones and low MIC values in our research indicate that (**1**′) effectively arrests bacterial growth, likely by disrupting essential bacterial enzymatic functions. Supporting this claim, previous studies have shown that organomercury compounds can inhibit type IA topoisomerases, critical enzymes for DNA replication and transcription, resulting in the accumulation of DNA fragmentation products and subsequent cell death [66].

The findings suggest that nanoparticle (**1**′) demonstrates strong antibacterial properties against a wide range of bacterial strains, with notable inhibition noted in both Gram‐positive and Gram‐negative bacteria. The MIC and MBC values affirm its bactericidal capabilities, positioning it as a promising candidate for further antimicrobial applications. Nonetheless, additional research is required to evaluate its effectiveness against antibiotic‐resistant bacteria. In addition, future studies should explore whether stabilization approaches, similar to those utilized by Malarvizhi et al, could enhance the safety profile of our nanoparticle, potentially making it a practical candidate for antimicrobial therapy [61].

### 3.5. MTT Assay

**FIGURE 18:**
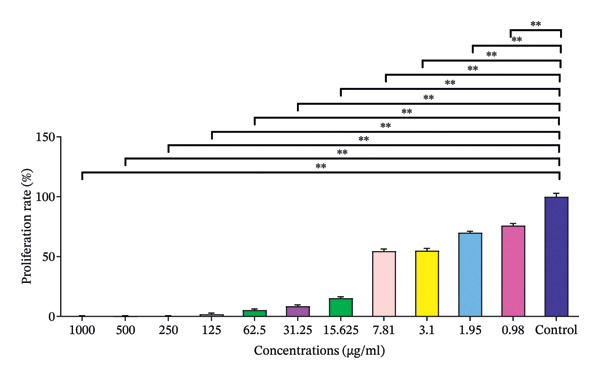
The treatment of OVCAR3 cells with compound (**1**′).

The cytotoxic effects of compound (**1**′) on OVCAR and Vero cell lines were investigated across various concentrations. In the OVCAR cell line, increasing nanoparticle concentrations demonstrated varying levels of cytotoxicity, as measured by mean cell viability percentages. Higher concentrations (1000 μg/mL and 500 μg/mL) showed lower viability with means of 0.67 ± 0.09 and 0.55 ± 0.16, respectively. A notable increase in cell viability was observed at lower concentrations, with values rising sharply from 5.44 ± 0.79 at 62.5 μg/mL to 75.96 ± 0.17 at 0.98 μg/mL. The control group consistently maintained 100% viability (Figure 18).

**FIGURE 19:**
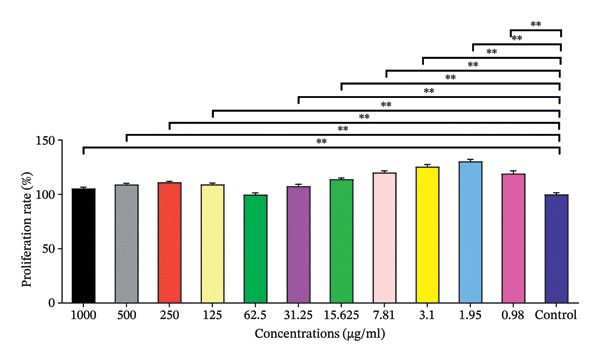
The treatment of VERO cells with compound (**1**′).

In contrast, Vero cells exhibited a different pattern of response to compound (**1**′). The highest concentration (1000 μg/mL) yielded a mean viability of 105.59 ± 1.03, and values slightly increased at intermediate concentrations, peaking at 125.65 ± 1.85 for 3.1 μg/mL. At 0.98 μg/mL, the viability decreased slightly to 119.26 ± 2.49, suggesting that Vero cells are less sensitive to (**1**′), compared with OVCAR cells (Figure 19).

**FIGURE 20:**
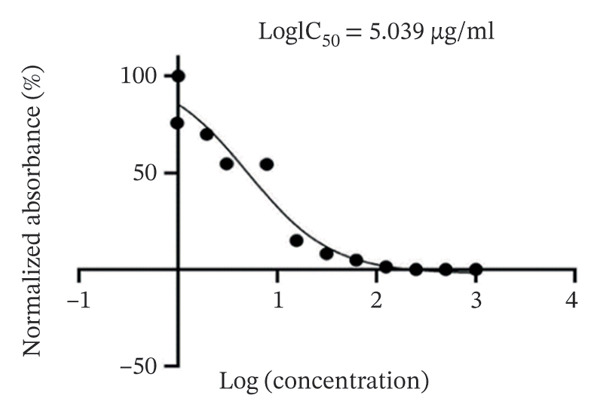
Cytotoxicity dose‐dependent curve of (**1**′) against OVCAR3 ovarian cancer cells.

The potency of (**1**′) against OVCAR3 ovarian cancer cells was quantified by calculating the half‐maximal inhibitory concentration (IC50). This was accomplished by performing an MTT‐based cytotoxicity assay to generate a dose‐response curve. The data on cell viability were subsequently fitted to a four‐parameter logistic (4PL) nonlinear regression model using GraphPad Prism (v8.4.3) to determine the IC50. The final value of 5.039 μg/mL represents the mean derived from three biologically independent replicates, each including technical triplicates (Figure 20).

These results indicate a dose‐dependent cytotoxic effect of (**1**′), on the OVCAR cell line, with lower concentrations showing reduced cytotoxicity. However, the Vero cell line displayed relatively higher resilience, with minimal decreases in cell viability even at lower concentrations. This suggests differential sensitivity between the two cell lines, highlighting the selective cytotoxic potential of (**1**′), in ovarian cancer cells. In medical treatments, using extremely high drug concentrations is generally avoided because they can be toxic to cells. Instead, the goal is to find the ideal dose that works effectively while causing minimal harm to healthy cells.

## 4. Conclusion

A new 1D mercury‐based CP was fabricated using two different methods: a layering technique to grow single crystals [(μ_2_‐Cl)(Ina)Hg(μ_3_‐Cl)Hg(μ_2_‐Cl)_2_(Ina)]_n_ (**1**) and a sonochemical process to produce nanoparticle (**1**′). Compound (**1**) contains two mercury centers, each with a different coordination number. In the crystal structure, two identical ligands are present, one attaches to the metal through its nitrogen atom, while the other connects via the oxygen atom in the carboxylate group. Additionally, a chlorine atom acts as a triple bridge, linking the two mercury centers (Hg1 and Hg2). The findings of biological activities suggest that nanoparticle (**1**′) demonstrates strong antibacterial properties against a wide range of bacterial strains, with notable inhibition noted in both Gram‐positive and Gram‐negative bacteria. The MIC and MBC values affirm its bactericidal capabilities, positioning it as a promising candidate for further antimicrobial applications. Nonetheless, additional research is required to evaluate its effectiveness against antibiotic‐resistant bacteria. This study highlights how using disk diffusion and microdilution methods together provides a more thorough way to evaluate the effectiveness of antimicrobial treatments, especially for complex formulations like nanoparticles. By combining these approaches, researchers can get a clearer picture of how well new antimicrobial agents work. The findings indicate that while mercury‐based composite nanoparticles show great promise as powerful antimicrobial agents, their potential toxicity and environmental impact need careful consideration. By improving their safety, we can not only make them more viable for therapeutic use but also strengthen our ability to tackle the growing global issue of antimicrobial resistance.

## Funding

No funding was received for this manuscript.

## Conflicts of Interest

The authors declare no conflicts of interest.

## Supporting Information

The crystallographic data for the structures in this study have been submitted to the Cambridge Crystallographic Data Centre (CCDC) as Supporting Information, under the reference number CCDC‐2354283.

## Supporting information


**Supporting Information** Additional supporting information can be found online in the Supporting Information section.

## Data Availability

The data that support the findings of this study are available from the corresponding authors upon reasonable request.
