# Supplementary material for: Synthesis, Topological, and Biological Studies of a Novel One‐Dimensional Hg(II) Coordination Polymer With Antibacterial and Anticancer Potentials
Source: Bioinorg Chem Appl. 2026 Jan 31;2026:2319593. doi: 10.1155/bca/2319593 (PMC12859819; doi:10.1155/bca/2319593)
Supplement: Supplementary file 1 — Supporting Information Additional supporting information can be found online in the Supporting Information section. [file BCA-2026-2319593-s001.pdf]

Title

Enter author details here

Abstract

Table 1

Experimental details

Crystal data	
Chemical formula	$C_6H_5Cl_2HgNO_2{\cdot}C_6H_5NO_2{\cdot}Cl_2Hg$
M_r	789.20
Crystal system, space group	Monoclinic, *Pc*
Temperature (K)	295
a, *b*, *c* (Å)	3.9045 (2), 18.5529 (12), 12.8519 (9)
β (°)	92.087 (5)
V (Å^3)	930.37 (10)
Z	2
Radiation type	Mo $K\alpha$
μ (mm^{-1})	17.07
Crystal size (mm)	0.28 × 0.12 × 0.10
Data collection	
Diffractometer	Xcalibur, Atlas
Absorption correction	Multi-scan<br>*CrysAlis PRO* 1.171.38.41 (Rigaku Oxford Diffraction, 2015) Empirical absorption correction using spherical harmonics, implemented in SCALE3 ABSPACK scaling algorithm.
T_{min}, T_{max}	0.791, 1.000
No. of measured, independent and observed [$I > 2\sigma(I)$] reflections	13815, 4419, 3105
R_{int}	0.062
$(\sin \theta/\lambda)_{max}$ (Å^{-1})	0.692
Refinement	
$R[F^2 > 2\sigma(F^2)]$, $wR(F^2)$, S	0.045, 0.059, 1.00
No. of reflections	4419
No. of parameters	218
No. of restraints	2
H-atom treatment	H-atom parameters constrained
$\Delta\rho_{max}$, $\Delta\rho_{min}$ (e Å^{-3})	0.83, −0.85
Absolute structure	Flack x determined using 1137 quotients [(I+)-(I-)]/[(I+)+(I-)] (Parsons, Flack and Wagner, Acta Cryst. B69 (2013) 249-259).
Absolute structure parameter	−0.027 (10)

Computer programs: *CrysAlis CCD* 1.171.38.43 (Rigaku Oxford Diffraction, 2015, *CrysAlis RED* 1.171.38.43 (Rigaku Oxford Diffraction, 2015), *SHELXT* 2014/5 (Sheldrick, 2014), *SHELXL2018*/3 (Sheldrick, 2018), Brandenburg & Putz (2006). Diamond 3.0. Crystal and Molecular Structure Visualisation, University of Bonn, Germany, *SHELXL2014*/7 (Sheldrick, 2014).

Acknowledgements

Funding information

References

Figure 1

supporting information

Title

Computing details

Data collection: *CrysAlis CCD* 1.171.38.43 (Rigaku Oxford Diffraction, 2015; cell refinement: *CrysAlis RED* 1.171.38.43 (Rigaku Oxford Diffraction, 2015); data reduction: *CrysAlis RED* 1.171.38.43 (Rigaku Oxford Diffraction, 2015); program(s) used to solve structure: *SHELXT* 2014/5 (Sheldrick, 2014); program(s) used to refine structure: *SHELXL2018*/3 (Sheldrick, 2018); molecular graphics: Brandenburg & Putz (2006). Diamond 3.0. Crystal and Molecular Structure Visualisation, University of Bonn, Germany; software used to prepare material for publication: *SHELXL2014*/7 (Sheldrick, 2014).

(A204)

Crystal data

$C_6H_5Cl_2HgNO_2{\cdot}C_6H_5NO_2{\cdot}Cl_2Hg$
M_r = 789.20
Monoclinic, *Pc*
a = 3.9045 (2) Å
b = 18.5529 (12) Å
c = 12.8519 (9) Å
β = 92.087 (5)°
V = 930.37 (10) Å^3
Z = 2
$F(000)$ = 712
D_x = 2.817 Mg m^{-3}
Mo $K\alpha$ radiation, λ = 0.71073 Å
Cell parameters from 2938 reflections
θ = 2.7–25.9°
μ = 17.07 mm^{-1}
T = 295 K
Parallelepiped, colourless
0.28 × 0.12 × 0.10 mm

Data collection

Xcalibur, Atlas diffractometer
Radiation source: fine-focus sealed X-ray tube
Detector resolution: 10.6249 pixels mm^{-1}
ω–scan
Absorption correction: multi-scan *CrysAlis PRO* 1.171.38.41 (Rigaku Oxford Diffraction, 2015) Empirical absorption correction using spherical harmonics, implemented in SCALE3 ABSPACK scaling algorithm.
T_{min} = 0.791, T_{max} = 1.000
13815 measured reflections
4419 independent reflections
3105 reflections with $I > 2\sigma(I)$
R_{int} = 0.062
θ_{max} = 29.5°, θ_{min} = 2.7°
$h = -4 \rightarrow 5$
$k = -24 \rightarrow 25$
$l = -17 \rightarrow 17$

Refinement

Refinement on F^2
Least-squares matrix: full
$R[F^2 > 2\sigma(F^2)]$ = 0.045
$wR(F^2)$ = 0.059
S = 1.00
4419 reflections
218 parameters
2 restraints
Primary atom site location: structure-invariant direct methods
Secondary atom site location: difference Fourier map
Hydrogen site location: inferred from neighbouring sites
H-atom parameters constrained
$w = 1/[\sigma^2(F_o^2) + (0.0078P)^2]$ where $P = (F_o^2 + 2F_c^2)/3$
$(\Delta/\sigma)_{max}$ = 0.002
$\Delta\rho_{max}$ = 0.83 e Å^{-3}
$\Delta\rho_{min}$ = −0.85 e Å^{-3}
Absolute structure: Flack x determined using 1137 quotients [(I+)-(I-)]/[(I+)+(I-)] (Parsons, Flack and Wagner, Acta Cryst. B69 (2013) 249-259).
Absolute structure parameter: −0.027 (10)

Special details

Geometry. All e.s.d.'s (except the e.s.d. in the dihedral angle between two l.s. planes) are estimated using the full covariance matrix. The cell e.s.d.'s are taken into account individually in the estimation of e.s.d.'s in distances, angles and torsion angles; correlations between e.s.d.'s in cell parameters are only used when they are defined by crystal symmetry. An approximate (isotropic) treatment of cell e.s.d.'s is used for estimating e.s.d.'s involving l.s. planes.

Refinement. Refinement of F^2 against ALL reflections. The weighted R-factor wR and goodness of fit S are based on F^2, conventional R-factors R are based on F, with F set to zero for negative F^2. The threshold expression of $F^2 > \sigma(F^2)$ is used only for calculating R-factors(gt) *etc*. and is not relevant to the choice of reflections for refinement. R-factors based on F^2 are statistically about twice as large as those based on F, and R- factors based on ALL data will be even larger.

Fractional atomic coordinates and isotropic or equivalent isotropic displacement parameters (Å^2)

	x	y	z	U_{iso}*/U_{eq}
Hg1	0.58248 (11)	0.26957 (4)	0.38389 (6)	0.0456 (2)
Hg2	0.23210 (13)	1.06990 (3)	0.48484 (6)	0.0455 (2)
Cl1	0.9245 (14)	0.2859 (3)	0.2440 (4)	0.0493 (14)
Cl2	0.2562 (12)	0.2491 (3)	0.5275 (4)	0.0500 (13)
Cl3	0.5650 (13)	1.1052 (3)	0.3426 (4)	0.0519 (14)
Cl4	−0.1456 (13)	1.0687 (3)	0.6238 (4)	0.0537 (17)
N1	1.145 (3)	0.4655 (7)	0.7543 (9)	0.037 (3)
H1	1.256876	0.462715	0.813046	0.045*
C2	1.076 (2)	0.5298 (5)	0.7147 (8)	0.047 (4)
H2	1.147005	0.571116	0.750396	0.056*
C3	0.897 (2)	0.5357 (5)	0.6199 (8)	0.045 (4)
H3	0.850874	0.580791	0.591185	0.054*
C4	0.790 (2)	0.4742 (4)	0.5684 (8)	0.031 (4)
C5	0.871 (2)	0.4093 (4)	0.6127 (8)	0.037 (4)
H5	0.806706	0.367009	0.578418	0.045*
C6	1.048 (2)	0.4055 (5)	0.7071 (8)	0.039 (4)
H6	1.098128	0.360962	0.737340	0.047*
C7	0.595 (2)	0.4780 (4)	0.4639 (8)	0.036 (4)
O1	0.552 (2)	0.4220 (4)	0.4141 (7)	0.049 (3)
O2	0.493 (2)	0.5402 (4)	0.4351 (6)	0.046 (3)
N11	0.306 (2)	0.9421 (4)	0.4621 (9)	0.046 (5)
C12	0.267 (2)	0.8967 (5)	0.5374 (9)	0.053 (5)
H12	0.196689	0.914257	0.600908	0.063*
C13	0.327 (2)	0.8211 (4)	0.5277 (8)	0.048 (5)
H13	0.301624	0.789954	0.583585	0.058*
C14	0.421 (2)	0.7969 (4)	0.4337 (8)	0.036 (4)
C15	0.458 (2)	0.8441 (4)	0.3535 (8)	0.046 (5)
H15	0.527261	0.827643	0.289279	0.056*
C16	0.391 (2)	0.9162 (5)	0.3682 (8)	0.052 (5)
H16	0.404894	0.947749	0.312235	0.062*
C17	0.491 (2)	0.7188 (5)	0.4145 (9)	0.040 (5)
O11	0.410 (2)	0.6761 (4)	0.4926 (8)	0.061 (4)
H11	0.432571	0.633870	0.475240	0.091*
O12	0.606 (2)	0.6953 (4)	0.3343 (8)	0.066 (4)

Atomic displacement parameters (Å^2)

	U^{11}	U^{22}	U^{33}	U^{12}	U^{13}	U^{23}
Hg1	0.0382 (3)	0.0481 (5)	0.0505 (4)	−0.0006 (4)	0.0034 (3)	−0.0007 (4)

Hg2	0.0437 (4)	0.0404 (4)	0.0530 (5)	0.0007 (4)	0.0121 (3)	−0.0010 (4)
Cl1	0.051 (3)	0.051 (3)	0.046 (3)	−0.004 (2)	0.007 (3)	0.001 (3)
Cl2	0.048 (3)	0.054 (3)	0.050 (3)	−0.002 (2)	0.013 (2)	−0.005 (3)
Cl3	0.055 (3)	0.049 (3)	0.054 (4)	0.000 (3)	0.019 (3)	0.000 (3)
Cl4	0.041 (3)	0.082 (4)	0.039 (3)	0.000 (2)	0.008 (2)	−0.004 (3)
N1	0.041 (7)	0.049 (10)	0.020 (7)	0.002 (7)	−0.011 (6)	−0.007 (7)
C2	0.063 (11)	0.028 (10)	0.047 (12)	0.004 (9)	−0.020 (9)	0.001 (9)
C3	0.060 (11)	0.031 (10)	0.043 (11)	−0.001 (8)	−0.021 (9)	0.007 (9)
C4	0.032 (8)	0.031 (10)	0.031 (10)	0.004 (7)	0.003 (7)	−0.011 (8)
C5	0.056 (10)	0.020 (9)	0.034 (10)	0.010 (8)	−0.013 (8)	−0.006 (8)
C6	0.055 (10)	0.031 (10)	0.031 (10)	0.001 (8)	0.000 (8)	0.009 (8)
C7	0.032 (8)	0.037 (11)	0.038 (11)	−0.001 (7)	0.004 (7)	0.003 (9)
O1	0.069 (8)	0.039 (8)	0.036 (8)	0.009 (7)	−0.025 (6)	−0.013 (6)
O2	0.079 (8)	0.026 (7)	0.031 (7)	0.006 (6)	−0.023 (6)	0.008 (6)
N11	0.048 (11)	0.039 (10)	0.054 (13)	0.000 (7)	0.012 (8)	−0.006 (9)
C12	0.073 (14)	0.043 (13)	0.043 (13)	−0.022 (11)	0.020 (11)	−0.004 (11)
C13	0.068 (12)	0.037 (12)	0.040 (12)	−0.006 (9)	0.005 (9)	0.007 (9)
C14	0.044 (9)	0.030 (10)	0.033 (10)	−0.005 (8)	−0.002 (8)	−0.017 (9)
C15	0.078 (13)	0.037 (12)	0.024 (10)	0.009 (10)	0.011 (9)	0.009 (9)
C16	0.061 (12)	0.056 (14)	0.039 (12)	−0.003 (9)	0.006 (9)	0.010 (10)
C17	0.054 (14)	0.030 (11)	0.037 (14)	−0.003 (9)	−0.010 (10)	0.009 (9)
O11	0.085 (9)	0.033 (8)	0.063 (9)	0.005 (7)	−0.012 (8)	−0.003 (7)
O12	0.091 (10)	0.042 (9)	0.067 (11)	0.004 (8)	0.029 (8)	−0.012 (8)

Geometric parameters (Å, º)

Hg1—Cl1	2.298 (5)	C6—H6	0.9300
Hg1—Cl2	2.312 (5)	C7—O1	1.230 (9)
Hg2—Cl4	2.357 (5)	C7—O2	1.270 (10)
Hg2—Cl3	2.373 (5)	N11—C12	1.300 (11)
Hg2—N11	2.407 (14)	N11—C16	1.350 (11)
Hg2—Cl4^{i}	2.963 (6)	C12—C13	1.430 (10)
N1—C6	1.317 (11)	C12—H12	0.9300
N1—C2	1.322 (11)	C13—C14	1.350 (11)
N1—H1	0.8600	C13—H13	0.9300
C2—C3	1.386 (11)	C14—C15	1.360 (11)
C2—H2	0.9300	C14—C17	1.500 (11)
C3—C4	1.376 (11)	C15—C16	1.380 (11)
C3—H3	0.9300	C15—H15	0.9300
C4—C5	1.365 (11)	C16—H16	0.9300
C4—C7	1.52 (2)	C17—O12	1.221 (11)
C5—C6	1.376 (11)	C17—O11	1.330 (11)
C5—H5	0.9300	O11—H11	0.8200
Cl1—Hg1—Cl2	177.3 (2)	O1—C7—O2	125.6 (15)
Cl4—Hg2—Cl3	164.02 (18)	O1—C7—C4	118.3 (15)
Cl4—Hg2—N11	99.4 (4)	O2—C7—C4	116.1 (7)
Cl3—Hg2—N11	96.1 (4)	C12—N11—C16	118.3 (16)
Cl4—Hg2—Cl4^{i}	93.75 (18)	C12—N11—Hg2	122.1 (12)
Cl3—Hg2—Cl4^{i}	90.35 (17)	C16—N11—Hg2	119.5 (13)
N11—Hg2—Cl4^{i}	88.1 (5)	N11—C12—C13	123.4 (18)
Hg2—Cl4—Hg2^{ii}	93.75 (18)	N11—C12—H12	118.3

C6—N1—C2	122.4 (13)	C13—C12—H12	118.3
C6—N1—H1	118.8	C14—C13—C12	117.0 (17)
C2—N1—H1	118.8	C14—C13—H13	121.5
N1—C2—C3	119.9 (15)	C12—C13—H13	121.5
N1—C2—H2	120.1	C13—C14—C15	120.1 (7)
C3—C2—H2	120.1	C13—C14—C17	121.7 (8)
C4—C3—C2	119.5 (15)	C15—C14—C17	118.2 (17)
C4—C3—H3	120.2	C14—C15—C16	119.7 (17)
C2—C3—H3	120.2	C14—C15—H15	120.2
C5—C4—C3	117.9 (15)	C16—C15—H15	120.2
C5—C4—C7	120.7 (14)	N11—C16—C15	121.4 (18)
C3—C4—C7	121.3 (7)	N11—C16—H16	119.3
C4—C5—C6	121.0 (15)	C15—C16—H16	119.3
C4—C5—H5	119.5	O12—C17—O11	122.1 (9)
C6—C5—H5	119.5	O12—C17—C14	124.1 (18)
N1—C6—C5	119.3 (14)	O11—C17—C14	113.8 (18)
N1—C6—H6	120.4	C17—O11—H11	109.5
C5—C6—H6	120.4		

Symmetry codes: (i) $x+1, y, z$; (ii) $x-1, y, z$.
